# A Study on the Grip Force of Ski Gloves with Feature Data Fusion Based on GWO—BPNN Deep Learning

**DOI:** 10.3390/s25237154

**Published:** 2025-11-23

**Authors:** Xiping Ma, Xinghua Gao, Yixin Zhang, Yufeng Gao

**Affiliations:** 1 School of Electrical and Information Engineering, Beihua University, Jilin 132021, China; 2 School of Mechanical Engineering, Beihua University, Jilin 132021, China

**Keywords:** hand grip strength, data fusion, GWO-BPNN algorithm

## Abstract

To investigate the characteristic pressure distribution patterns when gripping ski poles during skiing, this study addresses the challenges of measuring grip force on the complex curved surfaces of ski poles. A dataset of experimental samples was established, and grip force data were extracted using deep neural network (DNN) training. To reduce errors caused by dynamic force distribution and domain shifts due to varying hand postures, a hybrid method combining deep neural networks with the bio-inspired Gray Wolf Optimization (GWO) algorithm was proposed. This approach enables the fusion of hand-related feature data, facilitating the development of a high-precision grip force prediction model for skiing. A multi-point flexible array sensor was selected to detect force at key contact points. Through system calibration, grip force data were collected and used to construct a comprehensive database. A backpropagation (BP) neural network was then developed to process the sensor data at these characteristic points using deep learning techniques. The data fusion model was trained and further optimized through the GWO-BPNN (Gray Wolf Optimizer–backpropagation neural network) algorithm, which focuses on correcting and classifying force data based on dominant force-bearing units. Experimental results show that the optimized model achieves a relative error of less than 2% compared to calibration experiments, significantly improving the accuracy of flexible sensor applications. This model has been successfully applied to the development of intelligent skiing gloves, offering a scientific foundation for performance guidance and evaluation in skiing sports.

## 1. Introduction

In skiing, effective pole usage through proper force application is a critical factor in achieving optimal performance, closely associated with skiing posture. Hand grip strength serves as an important parameter for athlete guidance, training, and performance evaluation and is also a key metric in the ergonomic assessment of ski gloves [1,2]. Currently, most grip strength measurements rely on indirect methods, which fail to accurately capture the multi-dimensional characteristics of hand grasping forces. With advancements in technologies such as haptic feedback for robotic hands [3,4], multi-point flexible array sensors have been increasingly applied in both planar pressure distribution and curved-surface force detection. Compared to single-point sensor data fusion, flexible array sensors deployed on curved surfaces can provide more accurate multi-dimensional sensing data, thereby enhancing target detection performance [5,6,7]. Consequently, signal processing and analysis methods for flexible array sensors—particularly those that improve data accuracy—have become a focal point of research [8,9,10], with multi-dimensional data fusion algorithms drawing significant attention [11,12,13]. In the field of robotic grasp stability, K-nearest neighbor (KNN) algorithms applied to data from flexible array sensors have achieved classification accuracies exceeding 98% [13]. As a non-parametric, instance-based learning method, the KNN algorithm plays a crucial role in classification and regression tasks. However, it faces many challenges in practical applications, such as the selection of the optimal k value, computational efficiency, high-dimensional data processing, and sensitivity to noise and outliers [14]. To address these issues, researchers have proposed a series of improvement schemes. Minetal [15] utilized a deep encoder network to enhance the classification performance of KNN; Blau and Irani [16] constructed neural nearest neighbor blocks through continuous deterministic relaxation; Kasnesis et al. [17] developed Perception Net, a deep convolutional neural network for late sensor fusion; Różycki and Wolszlegera [18] compared the performance of neural networks and KNN classifiers in recognizing handwritten digits. Artificial neural networks (ANNs) have also been shown to reduce data errors in fusion processing of curved-surface multi-point array sensors [17,19]. Research on artificial intelligence applications provides new methods for data fusion of biometric features [20,21,22]. These findings demonstrate that compensating for non-target parameters and employing machine learning-based data fusion algorithms can yield relatively high performance. Among them, nonlinear neural network models have become a primary method for sensor data fusion. However, existing studies are mostly limited to local or planar surface applications. Research on data fusion for high-curvature surfaces, such as ski poles, remains limited, and the lack of publicly available datasets for training complex neural networks poses an additional challenge. As a result, lightweight neural network is needed.

Algorithms capable of learning from small samples are essential for addressing this issue.

Previous work has demonstrated that combining multilayer perceptions (MLPs) with Dynamic Time Warping (DTW) enables effective handwriting recognition and joint motion monitoring, while an SVM-MLP hybrid model applied to multimodal flexible electronic skins achieved an object recognition accuracy of 99.42% across 24 categories [23]. Neural network models have become the main means of sensor fusion, and deep learning methods such as convolutional neural networks have brought significant advantages to linear regression methods [20]. These results indicate that by adopting various optimization strategies, lightweight neural network models can attain high-accuracy sensor data fusion. This paper proposes a GWO-BPNN algorithm, which leverages the Gray Wolf Optimizer (GWO) to optimize the parameters of a backpropagation neural network (BPNN), targeting the integration of grip strength data acquired by flexible array sensors applied to high-curvature, small-area, multi-feature points on ski poles. The proposed method aims to support ergonomic evaluation of force and tactile feedback in ski gloves, enable intelligent monitoring of skiing posture and force application, and ultimately provide guidance for training and performance assessment.

## 2. Characterization of Skiing Hand Grip

**Figure 1:**
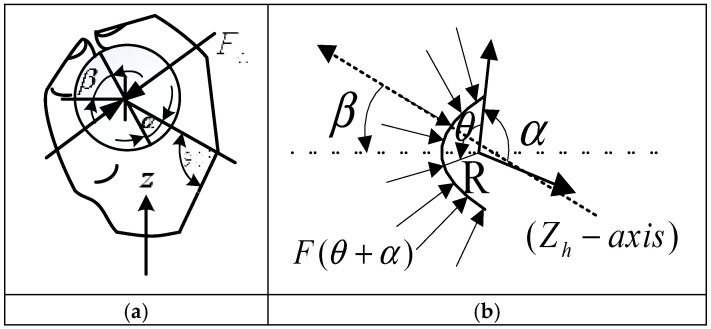
Illustration of the clamping force and grip direction. (**a**) Area of exposure and empowerment; (**b**) direction of grip force.

The process of skiing, which involves the use of ski poles, can be divided into a series of stages, starting with the “skating-support poles-propulsion” phase and culminating in the “grip-grip-hold” stage. This progression corresponds to the irregular curvature of the ski pole handle, characterized by significant variations in its contour. The hand and the grip area, which are in direct contact with the handle, play a critical role in stabilizing the skier’s body and maintaining control over the skis amidst these surface irregularities. The term “grip” refers to the clamping force exerted on a handle when the hand encloses it, while “grip force” is the clamping force acting on the handle in a closed grip. This force is counterbalanced by the clamping force exerted in the opposite direction along the division plane of the hand. The contact area and the distribution of the holding force are depicted in Figure 1a,b. Notably, the grip force exerted by the hand on the ski pole handle is not uniformly distributed [24]. Instead, its magnitude is typically a function of the reference axis or the division plane. In experimental studies on grip force measurement, the forearm along the Zh axis is often used as the reference axis, as shown in Figure 1b. The use of sensors to detect the forces exerted on the handle is a common approach. These sensors calculate the curvilinear integral of the forces distributed along the handle. By integrating the curves, i.e., $F_{N}(\alpha )=\iint_{s}f(\alpha +\theta )\sin(\theta )ds$, the omponents of the grip force are obtained. When the radius of handle is R, the grip force can be expressed mathematically as shown in Equation (1).

(1) $$F_{N}(\alpha )=R\int_{0}^{\pi }f(\alpha +\theta )\sin(\theta )d\theta$$

Grip force can be accurately measured using detection sensors, as demonstrated by Equation (1). However, these sensors often lack comprehensive coverage of the entire surface of the hand in contact with the grip area. Furthermore, the irregular shape of the ski pole handle adds complexity to detecting the total force components exerted by the hand. Given that the grip of hands involves contact at specific feature areas, this study adopts a feature-point detection approach. Small-area, flexible array pressure sensors are employed to measure the localized forces at these feature points. To address the challenges posed by incomplete coverage and complex force distributions, intelligent data fusion algorithms are utilized to integrate and analyze the sensor data, thereby overcoming the limitations of existing methods.

**Figure 2:**
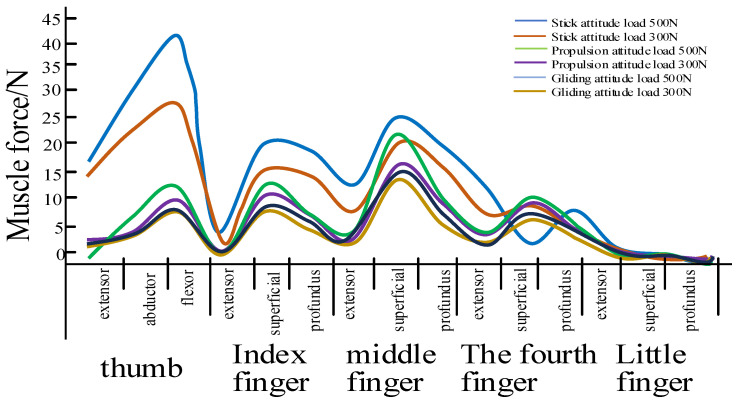
Multiple groups of hand muscles exerting force.

The forward power in skiing primarily comes from the propulsion provided by the ski poles. The muscle force exerted by the hand embodies the biodynamic characteristics of the hand, which can be analyzed using biomechanical modeling software such as AnyBody V6.0 [25,26,27,28,29,30]. This analysis focuses on the muscle force characteristics of the hand during skiing, particularly the relationship between hand loads and skiing posture [26]. Using Kinect bone-tracking technology [27,28], professional skiers were captured in three skiing positions: pole-supported, pushing, and gliding. A skiing model was then constructed, with human motion data collected from bone tracking. In MATLAB 2022b, the joint angles at key anatomical points under various skiing postures were calculated. Using skiing posture and hand load as research variables, a musculoskeletal model was established with the AnyBody Modeling System. Skiing states including pole planting, propulsion, and gliding were defined, and different loads were applied to the hands to simulate the force exertion during ski pole gripping. Biomechanical simulations were conducted to analyze muscle activation in the hand under various loading conditions. As illustrated in Figure 2, when a relatively high grip force of 500 N was applied—based on the national standard for adult grip strength [29]—the flexor pollicis longus exhibited a peak force of 39.70 N, and the flexor digitorum superficialis of the middle finger reached 25.80 N. The thumb and middle finger played a primary role in force generation, while the index and ring fingers contributed to auxiliary force. The little finger generated the least force, primarily serving to stabilize the hand posture during grip. Although variations in skiing posture and hand loading influence muscle activation levels, the proportional contribution of each muscle to the total grip force remains relatively stable across different force conditions.

**Figure 3:**
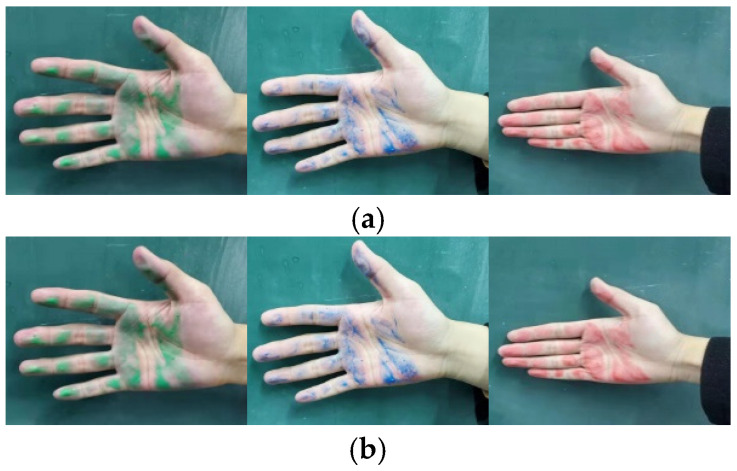

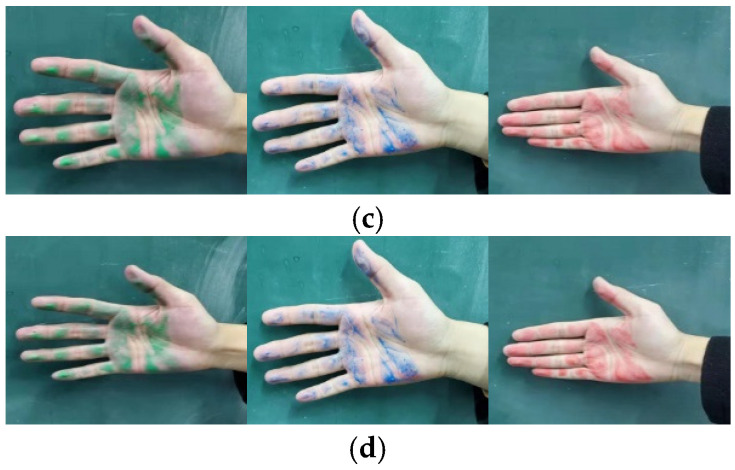
Characteristics of the distribution of hand grasping forms for Subjects 1 to 4. (**a**) Characteristics of the distribution of hand grasping modes for Subject #1; (**b**) Characteristics of the distribution of hand grasping modes for Subject #2; (**c**) Characteristics of the distribution of hand grasping modes for Subject #3; (**d**) Characteristics of the distribution of hand grasping modes for Subject #4.

To determine the main contact areas between the hand and the grip, three different pigment colors were applied. The hand-grip form test was conducted with four subjects using professional ski poles, each uniformly coated with a different pigment. During the test, the subjects simulated the correct skiing posture while gripping the handle of the ski poles, ensuring full contact with the handle for one minute. Afterward, the unfolded palms of the subjects were photographed, and the distribution of pigments and any areas without contact were analyzed. The resulting images are shown in Figure 3.

**Figure 4:**
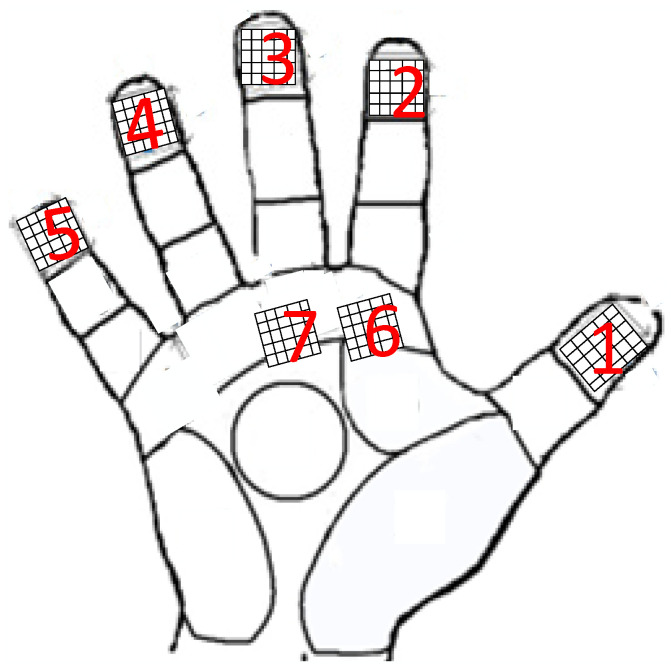
Flexible array pressure sensor mounting locations.

A comprehensive analysis of the comparisons shown in Figure 3a–d reveals that when the hand was grasping the ski pole, it was found that there were gaps in the palm area of the hand, and most of the joints of the hand were blank, indicating that these parts did not come into contact with the handle or had very little contact. Most of the hand joints also show no contact, suggesting that these regions do not contribute significantly to the grip. However, the tilt of the ski pole during the grip causes greater deformation in the thumb and middle finger areas, which increases the contact area between these regions and the ski pole grip. From this, it can be inferred that the primary muscle groups responsible for generating force during the grip are concentrated in the thumb and middle finger regions. The contact area is primarily concentrated around the thumb, middle finger, and the palm near the base of the fingers. The sensor attachment positions are shown in Figure 4, where the thumb is labeled as sensor No. 1, and the labels for the other sensor regions are also indicated in the figure.

## 3. Multi-Point Flexible Array Sensor Calibration Test Experiment

### 3.1. Hydraulic Handle Model and Test System

**Figure 5:**
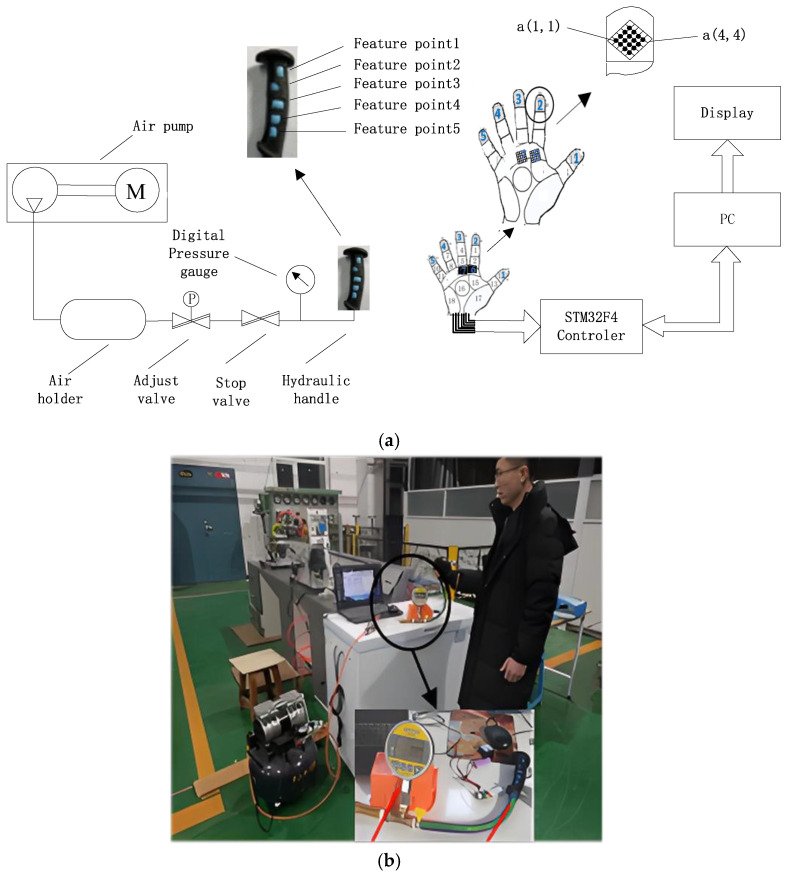
System design principal structure and test platform. (**a**) System design principles test platform; (**b**) hydraulic ski handles physical test system.

**Figure 6:**
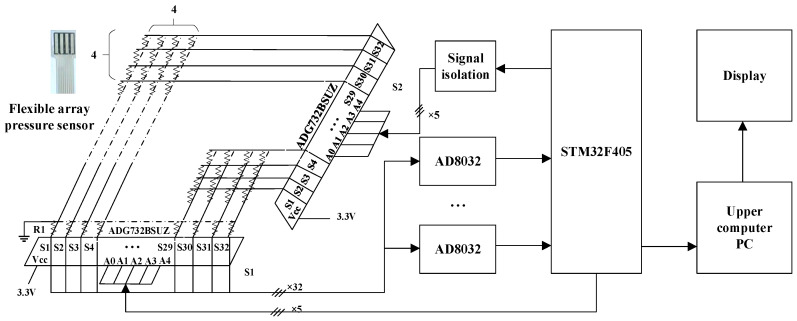
Hydraulic ski grip data acquisition system.

The ski poles used in the experiment were made from ALLOY7075, and a three-dimensional scanner was applied to create a 3D model of the handle. This model was then imported into 3D mapping software. Based on Figure 4, the force distribution characteristics of the hand gripping the ski pole were analyzed, with flexible array pressure sensors attached to the corresponding areas of the handle and the hand. The sensor’s sensing area was slightly larger than the corresponding area on the handle to ensure effective testing. A 3D-printed handle support shell was created to house the components. Inside the shell, a liquid-filled capsule was assembled, and the support shell was fitted to form a hydraulic handle. The pressure for the hydraulic handle was provided by an air pump, with an air pressure circuit connected to the hydraulic handle via a shut-off valve. The hydraulic circuit also included an MD-S200 intelligent digital pressure gauge. This setup formed the ski hand-grip test platform, as shown in Figure 5. The hydraulic ski grip data acquisition system, shown in Figure 6, was used to control the test system’s air pressure within the desired range. After closing the shut-off valve, the hydraulic handle maintained stable pressure in a sealed state, thus forming a capsule grip gauge. The pressure within the hydraulic handle was uniform, and the hand grip force was measured through the extrusion reaction of the hand grip [31,32].

In Figure 6, based on the gripping characteristic area of the hand and the grip strength standards for adult male hands [3], seven RX-M0404S model 4 × 4, 1 kg flexible array pressure sensors with dimensions of 14 × 14 mm^2^, and a test sensing area of 10 × 10 mm^2^ were selected. The performance specifications of the sensors are shown in Table 1. The flexible array pressure sensors are connected to a bus system, with each row of the bus directed through an analog switch (S2) connected to the computer. The column direction of the bus is grounded through a bias voltage divider resistor (R1), and an analog switch (S1) is used to connect the bus to the ADC for signal acquisition. The microcontroller controls S1 and S2: when S2 provides voltage to the *i*-th row of the bus, the microcontroller controls S1 to read the data from the *j*-th column, thereby obtaining the sensor voltage V*ij*. The data acquisition system synchronizes the sensor data using a multiplex sampler (ADG732BSUZ) for synchronous data collection. After multiplexing, the ADC0832 is used for analog-to-digital conversion under the control of the STM32F405 microcontroller. The data is then stored in an SD memory card and simultaneously uploaded to the PC through serial communication for display, storage, and post-processing.

### 3.2. Calibration Test

**Figure 7:**
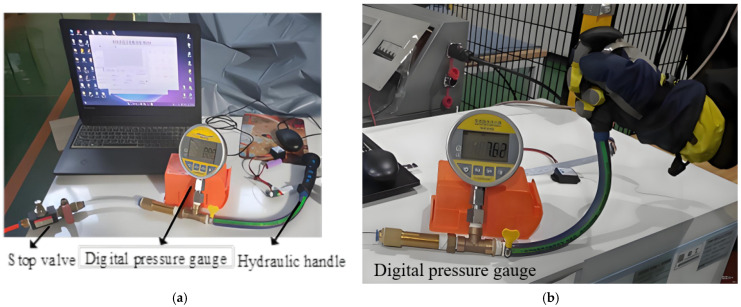
Hand-grip detection and hydraulic hand-grip force calibration experiment. (**a**) Hand-grip strength testing system; (**b**) Connection for hydraulic-handle calibration system.

The hand-grip strength test platform for skiing was used for hand-grip detection and the grip strength calibration of the hydraulic handle. The system was started, and the hydraulic system of the handle was adjusted to form a stable closed space for the grip strength test. Simulate the movement process of the ski poles, pushing and sliding the pressure of the hydraulic handle when grasping changes. Measure the pressure information data of the sensors in each characteristic area of the hand, record the total pressure under the corresponding pressure, and store it in the SD card of the controller as the calibration value of the dataset. The comprehensive hand force (grip force) was synchronously recorded. After extensive experiments on many subjects, the grip experiment data of each subject’s left and right hands were collected, respectively, and then packaged to form a file format that could be read and analyzed by MATLAB. In the test, considering the practical application, ski gloves were worn. The connection of the hand-grip strength detection system and the hydraulic handle-grip strength calibration system is shown in Figure 7.

### 3.3. Analysis of Output and Data Correction of Flexible Array Pressure Sensor

**Figure 8:**
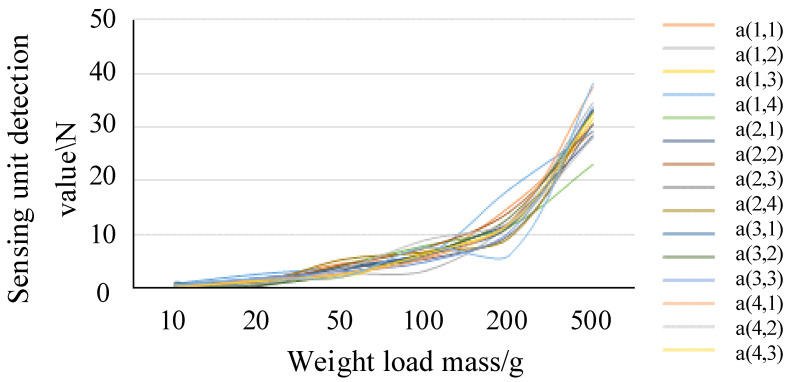
Characteristic curve of the detected voltage value in the unit sensing range.

To ensure the accuracy of the data collected by the grip force detection system, a static pressure verification test was conducted using standard weights with different masses as loads. Special shims were placed between the sensor and the load to improve pressure transfer efficiency. The test load ranged from 10 g to 500 g. The output results under six different loads. The characteristic curve of the load of the flexible sensor and the detected voltage values of each sensing area unit, after mean smoothing, is shown in Figure 8. From the above analysis, the detection outputs of each unit of the flexible array pressure sensor are nonlinear with the input, showing a positive correlation trend. There is a significant coupling phenomenon among the various sensing units. Moreover, when the curvature changes, the coupling phenomenon varies significantly with different forces, and it cannot meet the requirements for direct hand-grip force detection.

To accurately reflect the true value of the detected force, it is necessary to further calculate and verify the force of the single-chip flexible array pressure sensor. Due to the bending of the flexible sensor at the force characteristic points along the grasping surface, the force directions at each sensing unit point are different. The direction of the total force acting on the region is consistent with the normal direction of the maximum pressure detection point. The resultant force acting on the sensor is the sum of the normal components. Since the maximum pressure value is in dynamic change, in the 3D software, for the corresponding sensor test data of the handle model, the direction of the maximum force unit is selected, and the force is vertically projected onto the force surface to obtain the vertical force surface of the total force, so as to correct the output of each sensor and improve the accuracy.

**Figure 9:**
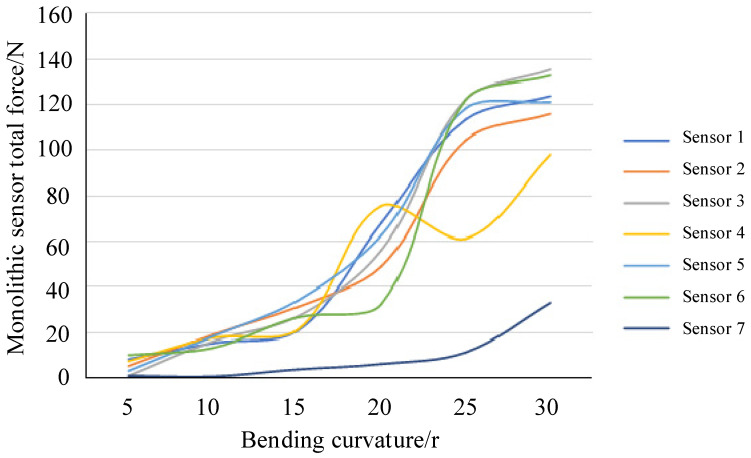
Force output curves for different characteristic areas.

According to the actual application, corresponding force outputs are obtained based on different bending curvatures, and after normalization processing, the curves are averaged and smoothed to obtain the curve shown in Figure 9. The nonlinearity of the force curve of a single sensor is significant, making it impossible to accurately calculate the force value. Considering the characteristics of the sensor and based on the large amount of data from the calibration test, a deep BPNN (BPNN) is adopted for training to study the data fusion model for obtaining the total force from each flexible array sensor. The accuracy of the data fusion is verified through model application.

## 4. Grip Fusion Algorithm Based on BPNN

### 4.1. Based on BPNN Grip Data Fusion Architecture

**Figure 10:**
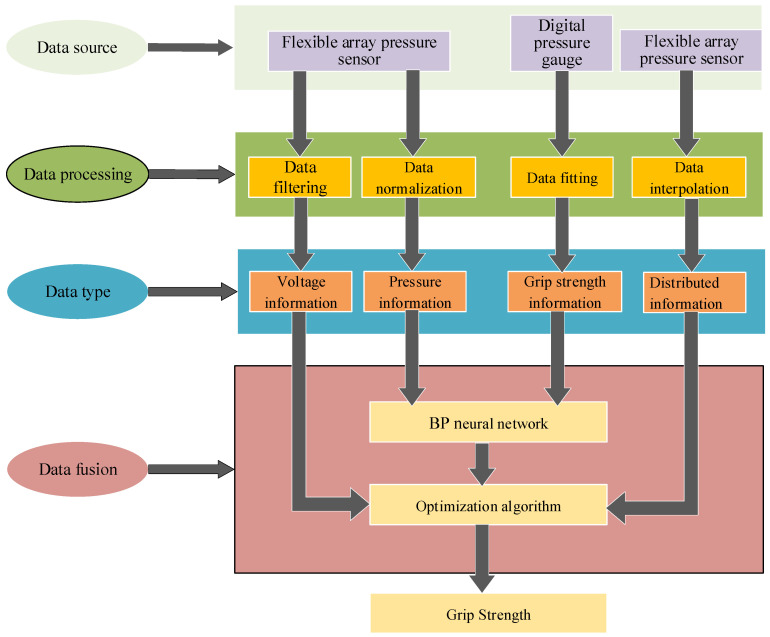
Grip data fusion architecture.

In the data fusion process, the test data from the ski-grip strength detection platform is used, where the labeled values correspond to the standard grip strength from the hydraulic grip detection system. These labeled values, along with the feature values from the flexible array pressure sensors, form the dataset for training the fusion model. Using MATLAB simulation software, a BPNN algorithm is applied to model and predict the grip strength of the skier’s hand while gripping the ski pole. The architecture of the grip-strength data fusion model is shown in Figure 10.

### 4.2. Mechanism of Data Fusion of Flexible Array Sensor Information for Hand Grip Feature Points

Utilizing the BPNN by forward information propagation and error backpropagation regulation principles, by designing the number of hidden layers, the output layer according to the same backpropagation is sent to the hidden layer and the input layer error of the decreasing slope is used to adjust each weight, until the network output error falls within the tolerance range or the specified training time is achieved.

For a given input vector *X*, the response of the network can be given by Y=T(X), where T is generally taken as a nonlinear operator related to the network structure. According to the calibrated experimental data, for a sample set of input and output vector pairs ($X_{p}$,$T_{p}$), *p* = 1, 2, ⋯, *N*; the adjustment of the weight array training is performed to meet the input when the output is *T_p_*, allowing the error index to achieve a very small value. *T_p_* denotes the desired output; *Y_p_* denotes the actual output of the current network; d denotes the distance function.

For the set of *N* samples, the performance metrics are

(2) $$E=\sum_{p=1}^{N}E_{p}=\sum_{p=1}^{N}\sum_{i=1}^{n_{0}}\phi (t_{pi}-y_{pi})$$

where ∅ is a positive definite, differentiable, and convex function.

For a unitary network with *n*_0_ outputs, the error function between each desired output vector *Tp* and the actual output vector *Yp* can be expressed as a sum of squared errors, i.e.,

(3) $$E_{p}=\frac{1}{2}\sum_{j=1}^{n_{0}}{\left(t_{pj}-y_{pj}\right)}^{2}$$

For each pair of input-output sample vectors (*X_p_*, *T_p_*), the computation of an intermediate vector *O_p_* must be included in the learning algorithm. According to the structure of the L-layer network, it is known that the output of the network is

(4) $$y_{pj}=\Gamma_{L}(Net_{pj}^{L})=\Gamma_{L}(\sum_{i=1}^{n_{L-1}}\omega_{ji}^{L}{ο}_{pi}^{\left(L-1\right)}+\theta_{j}^{L})\;j=1,2,\cdots ,n_{0}$$

The input of the *r* + 1 implicit layer is the output of the *r*th implicit layer, so that

(5) $${ο}_{pj}^{\left(r+1\right)}=\Gamma_{r+1}(\sum_{l=1}^{n_{r}}\omega_{jl}^{r+1}{ο}_{pl}^{r}+\theta_{j}^{r+1})\;r=0,1,2,\cdots ,L-1$$

When *r* = 0, $o_{pl}^{\left(0\right)}=x_{pl}$, and $n_{0}=n_{i}$.

The update of the training network’s weight array is realized by the error between the desired output and the actual output of the backpropagation network. From Equations (3)–(5), the performance metric function $E_{p}$ can be obtained:

(6) $$\frac{\partial E_{p}}{\partial \omega_{ji}^{r}}=\frac{\partial E_{p}}{\partial Net_{pj}^{r}}\frac{\partial Net_{pj}^{r}}{\partial \omega_{ji}^{r}}$$

(7) $$\frac{\partial Net_{pj}^{r}}{\partial \omega_{ji}^{r}}=\frac{\partial }{\partial \omega_{ji}^{r}}\sum_{k}\omega_{jk}^{r}{ο}_{pk}^{\left(r-1\right)}={ο}_{pi}^{\left(r-1\right)}$$

Definition: $\delta_{pj}^{r}=-\frac{\partial E_{p}}{\partial {\mathrm{Net}}_{\mathrm{pj}}^{r}}$; $\delta_{pj}^{r}$ is called the generalized error; then (6) and (7) can be written as follows: $\frac{\partial E_{p}}{\partial \omega_{ji}^{r}}=\delta_{pj}^{r}o_{pi}^{\left(r-1\right)}$. To reduce *E* according to the gradient, the weights must be adjusted according to the following equation:

(8) $$\Delta \omega_{ji}^{r}=\eta \delta_{pj}^{r}{ο}_{pi}^{\left(r-1\right)}$$

where the superscript *r* denotes the rth implicit layer, *r* = 1, 2, …, *L*; $\omega_{ji}^{r}$ is the connectivity coefficient from the *i*-th unit of the (*r*−1)th layer to the *j*-th unit of the *r*-th layer; *η* is the learning step.

### 4.3. Fusion of Data Information from Flexible Array Sensors for Hand Grasping Feature Points

**Figure 11:**
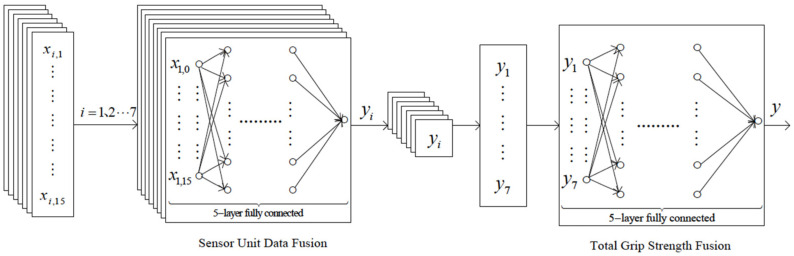
Architecture of the system BPNN model.

For each feature point, there are 16 input signals from individual sensors, generating one force output. The force direction at each sensing point on the sensor varies due to the curved surface. In the label experiment, the point with the maximum force was projected onto the vertical plane to obtain the total force effect as the actual output. Through theoretical calculation and the data fusion algorithm of neural network, the model relationship between the total force of a single sensor and each unit sensing area was trained. The experimental samples were selected from the teachers and students at the researcher’s university, as well as some skiers. The participants were aged between 18 and 50 years, and were divided into male and female groups, and professional and non-professional groups. The data were obtained through simulated actual movements tests, and the consistency of the test sample data was quite good. Randomly selecting 2400 sets of input and output data from the experimental dataset, 2200 sets were randomly chosen as the training data for the neural network, and 200 sets of random data were used as the test data to verify the predictive performance of the modeling network. The system model architecture is shown in Figure 11.

**Figure 12:**
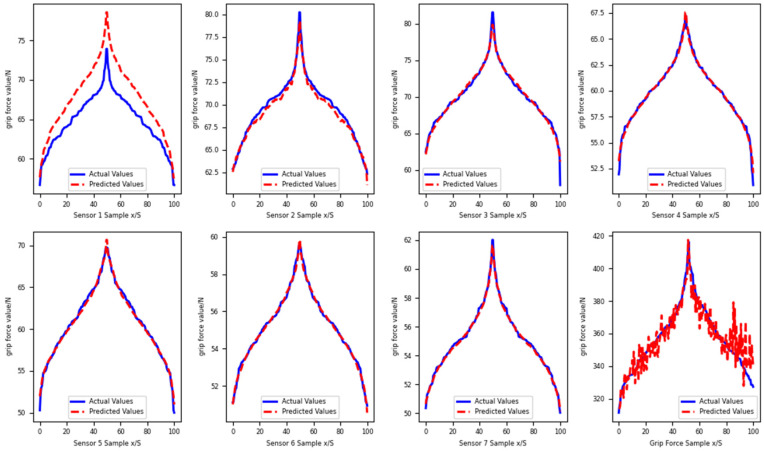
Fitting curves of the grasping feature points and the combined force BP model with the prediction set.

**Figure 13:**
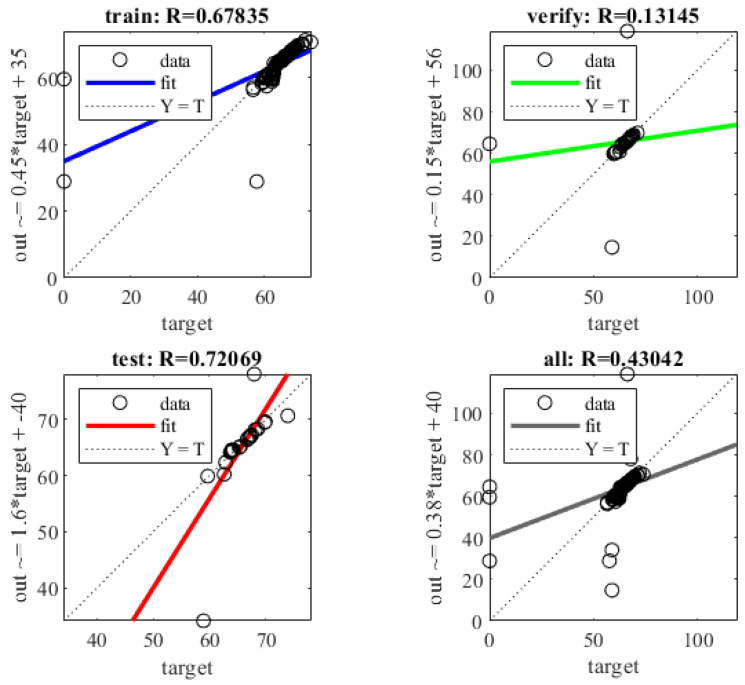
Regression results of BPNN.

Among them, the information of each feature point is used to train the model for the detection values of 16 sensing units of the single sensor using MATLAB, and a Π_16.512.256.128.64.1_ BPNN structure was constructed. A nonlinear sigmoid activation function was selected for model training and prediction. Furthermore, a Π_7.256.256.128.64.1_ network structure was constructed with an input of seven and an output of one, featuring four hidden layers, using the sigmoid activation function. The training target minimum error was set to 0.00001, the learning rate was 0.01, and the number of iterations was 500. The MATLAB model training and prediction for hand grasping grip force were obtained, and the fitting curves of the training model and actual prediction dataset for the seven feature point individual sensors, and the force training model and actual prediction dataset were obtained, as shown in Figure 12. The average relative error MAE of the test set of BPNN was 6.31%, the mean square error MSE was 38.4233%, and the root mean square error was 8.465%. The regression effect of BPNN for the right-hand grip force is shown in Figure 13. The R value of the training set is 0.4304, the R value of the validation set is 0.13145, and the R value of the test set is 0.72069.

The simulation results show that when the left hand grasps the ski pole handle, the predicted grip force value using the BPNN model does not closely match the actual grip force value. The predicted value does not change well in response to the change in grip force. The predicted grip force value in the fully grasped state deviates significantly from the actual grip force value. The maximum relative error in the BPNN grip force prediction results is 6.31%, the mean square error is 38.4233%, the root mean square error is 8.465%, and the average relative error is 6.584%.

From the above analysis, it can be concluded that the hand grip force prediction model based on BPNN cannot effectively predict the hand-grip force. During the process of grasping the ski pole handle with the hand, each local force application is different, and the total grip force is also different. Due to the influence of various factors such as the grip state of the hand to the fully grasped state and the force exerted by the hand muscles, the predicted grip force data of hand grasping will have a large error.

## 5. Optimized Grip Fusion Algorithm Based on GWO-BPNN

An analysis of the BPNN hand-grip strength prediction results reveals certain limitations, including significant errors in some prediction points. The main reasons for the changes in the force core points of the sensor are the differences in hand shape, ski poles, grasping, etc. It is necessary to update the core action points in real time and adjust the influence based on the maximum value in the input actual data and by level to improve the accuracy of the model. The GWO algorithm is selected as the optimizer for the research. To address these issues, the GWO (Gray Wolf Optimization) algorithm is used to optimize the BPNN. GWO is a bio-inspired optimization algorithm that mimics the hunting behavior of gray wolves, which cooperate and divide tasks when hunting. In this approach, the state of the artificial wolf represents the weights and thresholds of the BPNN [33]. The optimal weights and thresholds are determined as initial values through the process of information exchange and task allocation within the wolf pack. Since these initial values are derived from the wolf pack optimization algorithm, rather than from artificial estimates based on experience, this method is more accurate and rigorous than traditional prediction models [34,35].

### 5.1. GWO-BPNN Algorithm

Based on the three phases of the gray wolf pack predation process—encirclement, hunting, and attacking—the wolves are organized into hierarchical levels, typically consisting of the *α*, *β*, *δ*, and *ω* layers. Each layer has a distinct division of labor. Once the location of the prey is determined, the gray wolves begin to encircle its position vector:

(9) $$\vec{D}=\left|C\vec{\left(X_{p}\right)}\vec{\left(t\right)}-\vec{X(t)}\right|$$

(10) $$\vec{X}(t+1)=\vec{X_{P}}(t)-\vec{A}\vec{D}$$

where $\vec{A}$ and $\vec{C}$ are vectors of coefficients, ${\vec{X}}_{p}$ is the position vector of the prey, and ${\vec{X}}_{p}$ denotes the position vector of the gray wolf. *t* is the number of iterations, *X*(*t*) is a gray wolf, *X*(*t* + 1) is the next position it reaches, and *X_p_*(*t*) specifically refers to one of  α, β, or δ. The α layer is the lead wolf, the β and δ layers are two wolves below α, and the others are the bottom layers. The coefficient vectors $\vec{A}$ and $\vec{C}$ are represented as follows:

$$\vec{A}=2\vec{a}\vec{\left(r_{1}\right)}-\vec{a}$$

$$\vec{C}=2\vec{r_{2}}$$

where *r*_1_ and *r*_2_ are random vectors in [0, 1], and a is a decreasing value in [0, 2], usually α=2−2t/I (I is the maximum number of iterations). After encircling the prey, the gray wolf hunts the prey under the guidance of α, β, and δ.

Based on the dynamic changes in the action points of the hand grasping the walking stick, the model is constructed using the GWO-BPNN algorithm. This algorithm randomly mediates updates to the maximum action point of the core model and iteratively performs a global search to achieve the desired goal, thus improving the model’s accuracy. The process involves several steps, including the initialization of parameters, scent concentration determination, detection of wandering wolves, the approach of ferocious wolves towards the prey, and updates to the position of both the besieged wolves and the lead wolf.

1. Initialization of parameters, including the number of wolves, the maximum number of iterations, the wolf pack scale factor, the maximum number of trips, the distance determination factor, the step size factor, and the update scale factor. These parameters include the distance determination coefficient, the step length coefficient, and the update scale factor.
2. Determination of the odor concentration function: This method simultaneously computes two values—one expected and one predicted. These two values are generally not equal, and the odor concentration function is defined as the sum of the absolute errors between the expected *O_i_* and predicted values *Y_i_*.
3. Detecting wolf patrol behavior. As the most crucial member of the pack, the position of the lead wolf plays a vital role. It is necessary to iteratively evaluate the odor concentration *Y_i_* of the *i*-th detective wolf and the *Y*_lead_ of the lead wolf. Following this, the next steps are performed:
  (11) $$x_{id}^{p}=x_{id}+\sin(2\pi p/h)step_{a}^{d}$$
  In Equation (11), *step_a_* is the length of the wander.
4. Ferocious wolves approach their prey. In Equation (11), if *Y_i_* > *Y*_lead_, a ferocious wolf can replace the alpha wolf; if *Y_i_* < *Y*_lead_, the ferocious wolf continues to approach its prey, until *d*_is_ ≤ *d*_near_. The distance between wolf individuals *a* and *b* is defined using Manhattan distance, which is given by:
  (12) $$L(a,b)=\sum_{d=1}^{D}\left|x_{ad}-x_{bd}\right|d_{new}=\frac{1}{D_{gw}}\sum_{d=1}^{D}\left|\max_{d}-\min_{d}\right|$$

The variable *d* to be optimized lies within the range [*d*_min_, *d*_max_]. The value of *step_b_* represents the size by which the wolf approaches the lead wolf.

(13) $$x_{id}^{k+1}=x_{id}^{k}+step_{b}^{d}g\left(g_{d}^{k}-x_{id}^{k}\right)/\left|g_{d}^{k}-x_{id}^{k}\right|$$

5. Using the above steps, the status of the besieged individual wolf is updated.
  (14) $$x_{id}^{k+1}=x_{id}^{k}+\lambda gstep_{b}^{d}g\left|G_{d}^{k}-x_{id}^{k}\right|$$
  where λ is a random number between [−1, 1], and *step_b_* is the siege step of the first wolf.

6. According to the survival mechanism of the *t*-test, the position of the lead wolf should be continuously updated. This ensures that the entire wolf pack can be updated accordingly, maintaining overall consistency.
7. The state of the artificial wolf represents the weights and thresholds of the BPNN. The optimal weights and thresholds are determined as initial values through information exchange and responsibility allocation among the wolves. After selecting the initial values in the previous steps, the BPNN is trained to achieve the desired prediction performance.

### 5.2. GWO-BPNN Optimization Algorithm Flow

**Figure 14:**
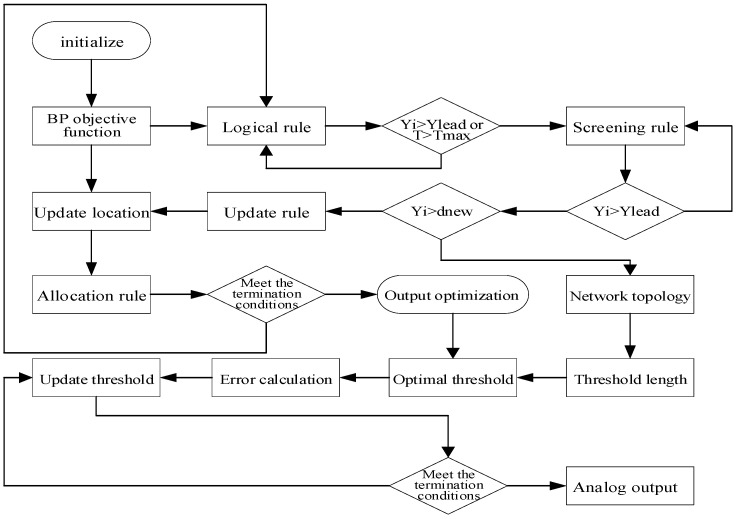
MATLAB simulation model flow for GWO-BPNN prediction.

The maximum value of the detection data from each flexible sensor at the feature point is taken as the core. The detection force at each feature point is updated and adjusted accordingly. The sensor data is classified, and software is used to correct the projected area of the force on the cane surface corresponding to each feature point. The GWO algorithm is then optimized with the BPNN expected output as the objective function. This process results in the development of the GWO-BPNN model for grip force prediction. The flowchart of this MATLAB simulation model is shown in Figure 14.

### 5.3. GWO-BPNN Algorithm MATLAB Modeling Training

**Figure 15:**
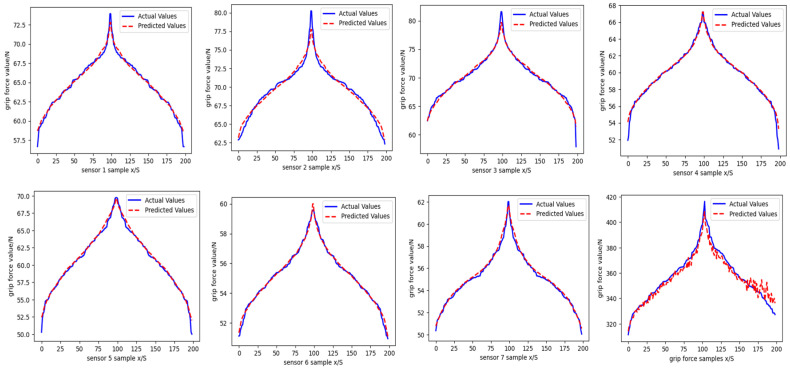
Fitting curves of the grip feature points and the combined force GWO-BPNN optimization model for the prediction set.

**Figure 16:**
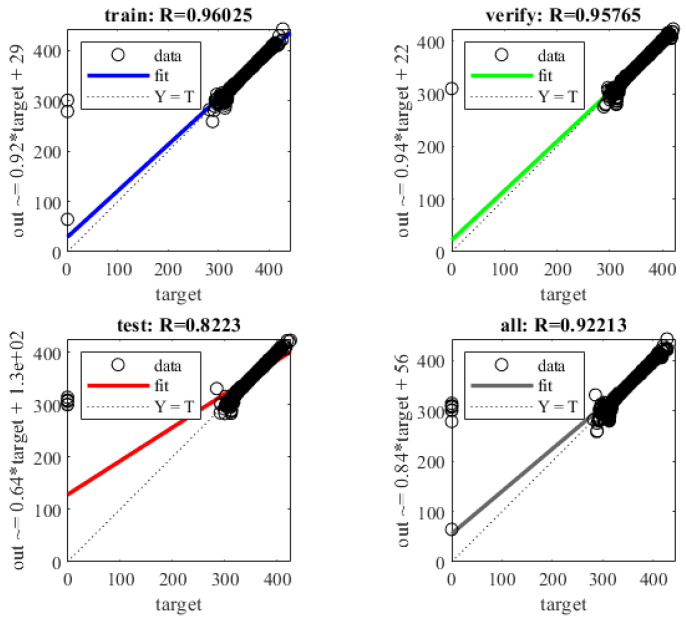
GWO-BPNN regression results.

The model parameters are set as follows: the population size is 20, the maximum number of iterations is 20, and the weight threshold boundary is [−5, 5]. The GWO-BPNN hand grip prediction model is established using MATLAB, and grip prediction is performed based on the process outlined in Figure 15. Optimization training using the GWO-BPNN algorithm is applied to the data from seven feature-point sensors, as well as the total hand grip force. The fitting curves of the training model and the actual prediction dataset, along with those for the ensemble training model, are shown in Figure 15. The regression results for the right-hand grip strength using the GWO-BPNN model are presented in Figure 16, with a regression R-value of 0.92213 for the training set, 0.95765 for the validation set, and 0.8223 for the test set.

Simulation results show that for the left hand gripping the ski pole, the predicted grip strength values using the GWO-BPNN network model closely match the actual values. The predicted values accurately follow changes in grip strength, with minimal deviation between the predicted and actual values in the tightly gripped state. The maximum relative error in the GWO-BPNN grip strength prediction is 1.25%, the mean square error is 0.95%, the root mean square error is 1.22%, and the average relative error is 1.54%. These errors are all below 2%, meeting the accuracy requirements for hand-grip strength prediction.

## 6. Grip Strength Test Experiment

**Figure 17:**
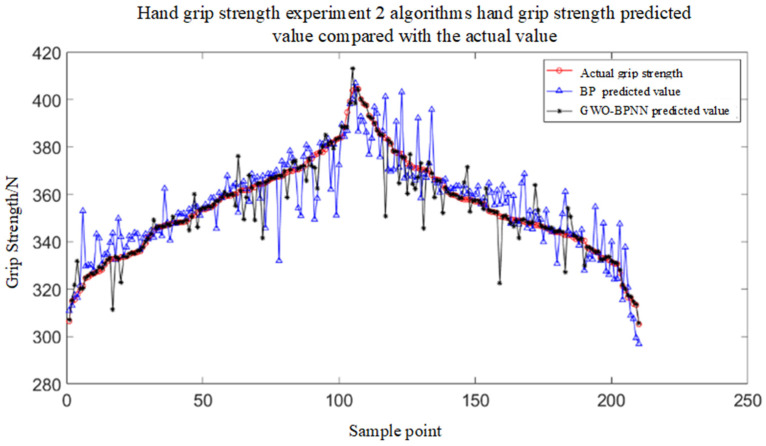
Comparison of predicted and actual values of grip strength between the 2 algorithms for hand-grip strength experiments.

The Gray Wolf Optimization (GWO) algorithm is applied to optimize the BPNN, resulting in the GWO-BPNN grip force prediction model. MATLAB is used to develop the serial port acquisition function, which connects to the GWO-BPNN grip force prediction model. The flexible array pressure sensor hand-grip force detection system is employed for real-time hand-grip force measurement, with the test platform as shown in Figure 5. Using the skiing hand-grip force detection platform, the subject wears flexible array pressure sensor-based grip force detection gloves and grasps the ski pole handle. The system records the detection values and predicts the grip force in real time using both the BPNN model and the GWO-BPNN model. The comparison of prediction results is shown in Figure 17.

It can be clearly seen from the figure that the comparison between the predicted values of the two algorithms and the actual grip force detection values shows that the predicted values of the GWO-BPNN grip force prediction model are closer to the actual grip force values, significantly higher than the BPNN grip force prediction method, and the improvement effect is obvious. The simulation results of the two algorithms are shown in Table 2. It can be known that the results obtained from the instance analysis of grip force prediction using the BP neural network and the GWO-BPNN model are that the grip force prediction accuracy of the GWO-BPNN model is significantly higher than that of the BP neural network method.

The simulation sample data for the above algorithm were collected from multiple adult individuals. After denoising and correcting, the sample data exhibit diversity and high accuracy. Despite variations in data distribution caused by individual differences, adjustments based on dynamically identified maximum dominant core force points enhance the model’s generalizability. However, for skiers with significantly smaller or larger hands, the variation in actual feature data is substantial, leading to relatively higher prediction errors and revealing certain limitations. Moreover, the system model has a relatively simple structure with limited parameter types, and model training converges within 20 iterations. Expert verification was used to achieve professional evaluation. The model gradually converges after 6 to 10 iterations during training and achieves better accuracy within 20 iterations.

## 7. Conclusions

This paper analyzes the gripping characteristics of the ski pole handle and uses multi-point flexible array sensors to detect the characteristic areas. It proposes an algorithm for obtaining grip force by fusing multiple detection data of curved surfaces using artificial intelligence. Through the performance test of the sensors, a calibration experimental system was developed. A prediction model for grip force was constructed using the BP neural network, and the model was optimized by the GWO-BPNN algorithm to improve the accuracy of grip force data fusion. The relative error is less than 2%, making the detection accuracy of the multi-point flexible array sensors more accurate. Based on the research of artificial intelligence data fusion, this paper provides a new application scheme for the detection of multi-point flexible array sensors, and offers new methods for the processing of force-tactile data in machine hands, sports such as skiing, medical intelligent equipment, etc. It improves the level of force-tactile detection and application. However, the accuracy of the research method in this paper depends on the accuracy of the calibration system and the reliability of the model. The testing accuracy of the sensors can be further improved by increasing the test accuracy of the experimental system and the sample size of the experimental data. To address the existing problems, test data samples of hand size, occupation, public, different levels of skiers, and different types of ski poles were added on the platform for training and improvement to enhance the generalization performance of the model, and it was used for real-time monitoring in the field, dynamic adjustment, and optimization. This paper mainly studies methods to improve the accuracy of grip force by fusing multi-dimensional data information of the grip handle. The problem of sensor coupling and nonlinearity is the follow-up content of this research, and this issue will be further explored in subsequent studies.

## Figures and Tables

**Table 1:** M0404S performance parameter table.

**Parameter**	**Value**	**Unit**	**Unit**
static resistance	>1	ΜΩ	depends on the range
repeatability	±8	%	physical property
operating voltage	3.3–5	V	it depends
temperature	−50–+60	℃	high temperature drift
response time	<20	ms	physical property

**Table 2:** Comparison of results between BPNN algorithm and GWO-BPNN algorithm.

**Object**	**Algorithm**	**Test Set R**	**Root Mean Square Error**	**Mean Relative Error**	**Maximum Relative Error**
right hand	BPNN	0.72069	8.465%	6.584%	6.31%
	GWO-BPNN	0.92213	1.22%	1.54%	1.25%

## Data Availability

The data supporting the findings of this study are openly available in a public repository. The complete dataset can be accessed at: https://github.com/WangChao0409/-sensors-3440169--raw-data.git (Accessed on 11 November 2025).
